# How do Rumination and Social Problem Solving Intensify Depression? A Longitudinal Study

**DOI:** 10.1007/s10942-017-0272-4

**Published:** 2017-05-08

**Authors:** Akira Hasegawa, Yoshihiko Kunisato, Hiroshi Morimoto, Haruki Nishimura, Yuko Matsuda

**Affiliations:** 1 Faculty of Human Relations, Tokai Gakuin University, 5-68 Naka-kirino, Kakamigahara City, Gifu 504-8511 Japan; 2 Department of Psychology, School of Human Sciences, Senshu University, 2-1-1, Higashimita, Tama-ku, Kawasaki-shi, Kanagawa 214-8580 Japan; 3 Faculty of Psychology, Meiji Gakuin University, 1-2-37 Shirokanedai Minato-ku, Tokyo, 108-8636 Japan; 4 Graduate School of Comprehensive Human Sciences, University of Tsukuba, 1-1-1 Tennodai, Tsukuba, Ibaraki 305-8577 Japan; 5 Faculty of Education, Hirosaki University, 1 Bunkyo-cho, Hirosaki, Aomori 036-8560 Japan

**Keywords:** Depression, Rumination, Social problem solving, Avoidance, Impulsivity, Stress generation

## Abstract

In order to examine how rumination and social problem solving intensify depression, the present study investigated longitudinal associations among each dimension of rumination and social problem solving and evaluated aspects of these constructs that predicted subsequent depression. A three-wave longitudinal study, with an interval of 4 weeks between waves, was conducted. Japanese university students completed the Beck Depression Inventory-Second Edition, Ruminative Responses Scale, Social Problem-Solving Inventory-Revised Short Version, and Interpersonal Stress Event Scale on three occasions 4 weeks apart (*n* = 284 at Time 1, 198 at Time 2, 165 at Time 3). Linear mixed models were analyzed to test whether each variable predicted subsequent depression, rumination, and each dimension of social problem solving. Rumination and negative problem orientation demonstrated a mutually enhancing relationship. Because these two variables were not associated with interpersonal conflict during the subsequent 4 weeks, rumination and negative problem orientation appear to strengthen each other without environmental change. Rumination and impulsivity/carelessness style were associated with subsequent depressive symptoms, after controlling for the effect of initial depression. Because rumination and impulsivity/carelessness style were not concurrently and longitudinally associated with each other, rumination and impulsive/careless problem solving style appear to be independent processes that serve to intensify depression.

## Introduction

Depressive rumination is defined as “behaviors and thoughts that focus one’s attention on one’s depressive symptoms and on the implications of these symptoms” (Nolen-Hoeksema 1991, p. 569). Previous experimental studies have shown that inducing rumination increases negative mood in dysphoric participants (Nolen-Hoeksema and Morrow 1993; Lyubomirsky and Nolen-Hoeksema 1995; Lyubomirsky et al. 1999; Lavender and Watkins 2004). Higher total scores on the Ruminative Responses Scale (RRS; Nolen-Hoeksema and Morrow 1991) have been found to be predictive of more severe depression (Nolen-Hoeksema and Morrow 1991; Hasegawa et al. 2013) and the onset of major depressive episodes (Nolen-Hoeksema 2000; Spasojević and Alloy 2001). Two factors that have been extracted from the RRS include *brooding*, which involves “a passive comparison of one’s current situation with some unachieved standard,” and *reflection*, which is “a purposeful turning inward to engage in cognitive problem solving to alleviate one’s depressive symptoms” (Treynor et al. 2003, p. 256). Several longitudinal studies have shown that brooding was associated with more depression at 6 months to 1 year later, whereas reflection was associated with less depression or showed no such association, indicating that brooding may represent a more maladaptive aspect of rumination than reflection (Treynor et al. 2003; Pearson et al. 2010; Schoofs et al. 2010; but see Hasegawa et al. 2015a).

One possible reason why rumination or its brooding subcomponent exacerbate depression is the valence of these thoughts. Because the content of rumination is mainly negative in valence (Lyubomirsky et al. 1999), the persistence of these negative thoughts increases depression. It is plausible that the relationship to depression is weaker for reflection than for brooding because of reflection’s less negative valence (a sample item of the reflection subscale of the RRS is “Analyze recent events to try to understand why you are depressed,” while an item for brooding is “Think ‘Why do I have problems other people don’t have?’”). Furthermore, rumination may have a mutually enhancing relationship with other cognitive-behavioral processes, including interpretation, attention, memory, and inhibited instrumental behavior (Lyubomirsky and Tkach 2004). A vicious cycle between rumination and maladaptive cognitive-behavioral processes may intensify depression.

Social problem solving is clusters of responses that are assumed to be related with rumination (Nolen-Hoeksema 1991; Lyubomirsky et al. 1999; Hasegawa et al. 2015b). Social problem solving is the process by which people attempt to identify or discover effective and adaptive solutions to problems they experience in everyday life (D’Zurilla et al. 2002). Five dimensions of the social problem solving process have been proposed; these are evaluated in the Social Problem-Solving Inventory-Revised (SPSI-R; D’Zurilla et al. 2002), or a short version of the SPSI-R (SPSI-R:S; D’Zurilla et al. 2002): *Positive problem orientation* (appraising a problem as a challenge and as solvable; believing that successful problem solving takes time, effort, and persistence), *negative problem orientation* (viewing problems as a significant threat to wellbeing or as unsolvable and becoming frustrated and upset when confronted with problems in life), *rational problem solving* (finding effective solutions to problems in a way that involves rational, deliberate, systematic, and skillful application of specific problem solving tasks), *impulsivity/carelessness style* (attempting to solve a problem in an impulsive, hurried, and incomplete manner), and *avoidance style* (avoiding problems, procrastinating, and depending on others to solve one’s problems). Anderson et al. (2011) showed that negative problem orientation predicted higher levels of depression 3 months later, after controlling for baseline depressive symptoms, while Hasegawa et al. (2015a) showed that impulsivity/carelessness style was predictive of more severe depression after 6 months (Nezu et al. 2008, for review).

Previous studies have examined whether rumination and social problem solving are associated. For example, cross-sectional studies that used the RRS and SPSI-R or SPSI-R:S showed that in nonclinical samples, rumination and its brooding subcomponent were positively associated with negative problem orientation and avoidance style (McMurrich and Johnson 2008; Hasegawa et al. 2015b, 2016). These findings were consistent with experimental studies showing that induced rumination may lead to negative interpretations about the self, one’s situation, and future events (Lyubomirsky and Nolen-Hoeksema 1995; Lyubomirsky et al. 1999; Lavender and Watkins 2004). Negatively biased thinking caused by rumination may also occur in problem solving processes, leading to higher degree of negative problem orientation. These negative interpretations may activate avoidance goals, leading to an avoidance style that prevents a person from solving his or her problems. Such a problem solving style would likely work to degrade his or her environment, as well as generate sustained rumination about problems or about the self.

On the other hand, rumination and its subcomponents have been shown to be relatively independent of the impulsivity/carelessness style (*r*s ranging from .04 to .14; Hasegawa et al. 2015b, 2016). This is important because the impulsivity/carelessness style represents another important factor that predicts depression. Hasegawa et al. (2015a) conducted a stepwise multiple regression analysis with scores on the Beck Depression Inventory-Second Edition (BDI-II; Beck et al. 1996) and all subscales of the RRS and SPSI-R:S and effectiveness scores on the Means-Ends Problem-Solving Test (MEPS; Platt and Spivack 1975) as independent variables, and BDI-II scores assessed 6 months later as the dependent variable. Only baseline depression level and impulsivity/carelessness style predicted future depressive symptoms. These findings suggest the possibility that the impulsivity/carelessness style intensifies depression via a process that is independent of rumination. This result is consistent with many previous findings showing that impulsivity, which is assumed to be a superordinate construct of the impulsivity/carelessness style, is a psychological correlate and vulnerability factor for depression (Saddichha and Schuetz 2014; Wright et al. 2014; Berg et al. 2015).

Although previous findings have shown positive associations between rumination, negative problem orientation, and avoidance style, as well as very weak or no associations between rumination and the impulsive/careless problem solving style, previous cross-sectional studies were unable to indicate causality. Therefore, the present study endeavored to examine prospective associations between rumination and social problem solving. In order to examine how rumination and social problem solving work to intensify depression, we also investigated the dimensions of rumination and social problem solving that predict future depression.

We further examined whether depression, rumination, and social problem solving increase interpersonal stressors. Because previous studies have shown that individuals with high depressive symptomatology or maladaptive characteristics related to depression tend to generate stressful events (for a review, see Liu and Alloy 2010), it was plausible that the variables we assessed would be related to such stress generation processes. Such an examination could help clarify how depression, rumination, and social problem solving are related to one other. We used the frequency of interpersonal conflicts as an index of such stressors, based on the likelihood that these events were caused, at least in part, by participants’ behaviors.

In sum, the present study investigated longitudinal relationships among depression, rumination, and social problem solving in order to examine how rumination and social problem solving intensify depression. We conducted a three-wave longitudinal study with an interval of 4 weeks between waves. To estimate the stable predictive powers of each variable, linear mixed models were analyzed in which depression, rumination (assessed with the RRS total scale), and each dimension of social problem solving were treated as fixed factors, with the same scale or subscale assessed at the next time point as dependent variables (details and advantages of this analytic method are described in the Statistical Analysis section). Because previous cross-sectional studies indicated that rumination, negative problem orientation, and avoidance style are associated with one another (McMurrich and Johnson 2008; Hasegawa et al. 2015b, 2016), we predicted that these variables would also show longitudinal associations. Moreover, considering the observed very weak or absent correlations between impulsivity/carelessness style and rumination (Hasegawa et al. 2015b, 2016), we expected that impulsivity/carelessness style would not be predicted by rumination, and vice versa. Given the findings of previous research (Nolen-Hoeksema and Morrow 1991; Nolen-Hoeksema 2000; Spasojević and Alloy 2001; Anderson et al. 2011; Hasegawa et al. 2015a), we predicted that rumination, negative problem orientation, and impulsivity/carelessness style would predict increased depressive symptoms assessed 4 weeks later, even after controlling for initial depression levels. We did not have any specific hypotheses about the predictive powers of other dimensions of social problem solving (i.e., positive problem orientation and rational problem solving) due to lack of a clear theoretical basis for such predictions.

We repeated the same analyses after replacing RRS total scale with brooding or reflection subscales. The findings were expected to be same as those obtained using RRS total scale when predictor or dependent variables were replaced with brooding in the analyses. However, because reflection appears to be an adaptive, or less maladaptive aspect of rumination (Treynor et al. 2003), we expected that any longitudinal associations between reflection and depression, negative problem orientation, and avoidance style would be weaker than those for RRS total scale. Finally, we explored whether depression, rumination, or each of the dimensions of social problem solving increases the frequency of subsequent interpersonal stressors.

## Method

### Participants

Two-hundred and eighty-five Japanese undergraduate and graduate students took part in the first questionnaire administration (Time 1) in March 2015. Participants were recruited from the Hirosaki University, Hiroshima International University, Senshu University, Tokai Gakuin University, and University of Tsukuba in Japan, and completed a packet of questionnaires in a small group setting. Students that were under treatment by a psychiatrist, clinical psychologist, or a counselor were excluded from the study for ethical reasons, because of the possibility that their mood could deteriorate as a result of participation in the study.

At intervals of about 4 weeks (Time 2) and 8 weeks (Time 3) later, participants completed the same questionnaires as at Time 1. A researcher sent the questionnaire packet to the participants, and participants were asked to complete and return the questionnaires within 1 week. Two-hundred and two participants at Time 2 and 173 participants at Time 3 returned the questionnaires.

Of the data provided by 285 participants at Time 1, the responses of one individual were excluded because we suspected that the participant provided false responses to the questionnaires (e.g., step-like answers to successive items such as “1, 2, 3, 4, 3, 2, 1”). This participant’s scores at Time 2 were also excluded. Of the data from 202 participants at Time 2, one individual was excluded for the reason described above, and three were excluded because they completed the questionnaires more than 35 days after Time 1 (i.e., 4 weeks plus 1 week). Of the data from 173 participants at Time 3, eight were excluded because they completed the questionnaires more than 63 days after Time 1 (i.e., 8 weeks plus 1 week). The final samples comprised 284 individuals at Time 1, 198 at Time 2, and 165 at Time 3. The sample at Time 1 comprised 118 men and 166 women, and the mean age of the sample was 20.07 (*SD* = 2.50; range 18–43).

### Measures

#### Beck Depression Inventory-Second Edition (BDI-II; Beck et al. 1996)

The BDI-II is a well-validated 21-item self-report questionnaire that measures the severity of depressive symptoms experienced in the past 2 weeks. Participants rate their responses using a 0–3 scale, with higher scores indicating greater severity of depression. The Japanese translation by Kojima and Furukawa (2003) was used in this study. Average scores, *SDs*, and alpha coefficients for the BDI-II and the other measures at each time point are displayed in Table 1.

**Table 1:** Descriptive statistics for each scale at each time point

	*n*	*M*	*SD*	*α*
*Time 1*				
BDI-II	281	12.87	8.94	.89
Brooding	284	13.07	3.95	.78
Reflection	284	10.43	3.52	.70
RRS total	283	51.48	13.38	.91
Positive problem orientation	284	9.61	4.23	.79
Negative problem orientation	284	10.74	4.54	.80
Rational problem solving	283	9.69	4.02	.74
Impulsivity/carelessness style	284	6.16	4.06	.75
Avoidance style	283	6.71	4.24	.78
ISE	284	16.00	6.17	.87
*Time 2*				
BDI-II	195	11.30	8.93	.91
Brooding	197	12.78	3.92	.81
Reflection	197	9.89	3.47	.74
RRS total	195	49.71	13.04	.91
Positive problem orientation	196	9.46	4.35	.82
Negative problem orientation	197	10.77	4.68	.84
Rational problem solving	198	9.37	3.84	.77
Impulsivity/carelessness style	197	6.02	3.92	.78
Avoidance style	198	7.11	4.34	.83
ISE	197	15.22	5.53	.86
*Time 3*				
BDI-II	163	10.60	9.36	.92
Brooding	165	12.67	3.96	.82
Reflection	165	9.64	3.57	.80
RRS total	163	49.21	13.84	.93
Positive problem orientation	163	9.80	4.24	.82
Negative problem orientation	164	10.71	4.96	.88
Rational problem solving	162	9.41	4.21	.82
Impulsivity/carelessness style	163	6.42	4.14	.81
Avoidance style	163	7.33	4.34	.82
ISE	164	14.88	5.81	.88

*BDI*-*II* Beck Depression Inventory-Second Edition, *RRS* Ruminative Responses Scale, *ISE* Interpersonal Stress Event Scale

#### Ruminative Responses Scale (RRS; Nolen-Hoeksema and Morrow 1991)

The RRS includes 22 items, each of which is rated on a 4-point rating scale anchored between 1 (*almost never*) and 4 (*almost always*). The RRS consists of five items assessing brooding, five items assessing reflection, and 12 depression-related items. Brooding and reflection subscale scores and total RRS scores were calculated. Adequate psychometric properties of the RRS, including good internal consistency and construct validity, as well as moderate test–retest reliability for the total and subscale scores, have been reported (Treynor et al. 2003; Schoofs et al. 2010). The Japanese translation by Hasegawa (2013) was used in this study.

#### Social Problem-Solving Inventory-Revised Short Version (SPSI-R:S; D’Zurilla et al. 2002)

The SPSI-R:S is a 25-item self-report scale designed to measure an individual’s cognitive, affective, and behavioral responses to real life problem solving situations. Each item is rated on a 5-point rating scale anchored between 0 (*not at all true of me*) and 4 (*extremely true of me*). The scale is composed of five subscales that include positive problem orientation, negative problem orientation, rational problem solving, impulsivity/carelessness style, and avoidance style. The SPSI-R:S has demonstrated good reliability and validity (D’Zurilla et al. 2002). The Japanese translation by Hasegawa et al. (2015b) was utilized in this study.

#### Interpersonal Stress Event Scale (ISE; Hashimoto 1997)

The ISE is a reliable and valid measure composed of 30 items and that includes three subscales: interpersonal conflict (e.g., “I quarreled with my acquaintance,” “I was insulted by others,” and “I disagreed with my acquaintance”), interpersonal inferiority complex (e.g., “There was awkward silence during a conversation”), and interpersonal dislocation (e.g., “I talked with a person with whom I was not so intimate”). This scale assesses the frequencies of interpersonal stressors that Japanese young adults often encounter in daily life. Participants indicated how often they encountered each event over the past 4 weeks on a 4-point scale, with 1 indicating *not at all* and 4 indicating *often*. In order to assess deterioration of interpersonal relationships caused by participants’ behaviors, only the interpersonal conflict subscale (composed of 9 items) was used in the analyses.

### Procedure

At Time 1, all participants that agreed to take part in this study completed the packet of questionnaires that included the BDI-II, RRS, SPSI-R:S, ISE, and two other measures that were not related to the purposes of the present study, in a small group setting. The procedure took participants about 20 min to complete. Researchers sent out the same questionnaire packet so that the packet could reach each participants’ home about 25 days (Time 2) and 53 days (Time 3) from completion at Time 1. Participants were asked to complete and return the questionnaires to the researchers within 1 week. The average duration between administration of the questionnaire package at Time 1 and Time 2 for the final sample was 27.71 days (*SD* = 2.41; from 24 to 35 days), that between Time 2 and Time 3 was 28.33 days (*SD* = 2.98; from 20 to 38 days), and that between Time 1 and Time 3 was 55.78 days (*SD* = 2.57; from 53 to 63 days). All participants received a book voucher worth 500 yen (approximately US $4) at Time 1 and 1000 yen (approximately US $8) at Time 3 for their participation. The Ethics Committee of Tokai Gakuin University approved this study.

### Statistical Analysis

All analyses were performed using SPSS ver. 23. Raw scores for each variable were analyzed, and missing data was handled with a pairwise method. Zero-order Pearson’s correlations were computed between each of the measures. To examine the variables that predict depression, rumination, and each dimension of social problem solving assessed 4 weeks later, linear mixed models with maximum likelihood estimation were evaluated. Depression, rumination assessed with RRS total scale, and each dimension of social problem solving were analyzed as fixed factors, and each scale or subscale assessed at the next time point was used as a dependent variable. We evaluated a total of seven linear mixed models. For example, the first model tested whether rumination and each dimension of social problem solving assessed at Times 1 and 2 predicted depressive symptoms at Times 2 and 3, respectively, controlling for the effects of depressive symptoms at the prior assessment. In all models, the repeated covariance type was set as unstructured covariance.

These models tested the average predictive effect of the independent variable on the dependent variable over each of the 4-week intervals in the three-wave data set. This analytic method produces relatively stable longitudinal associations between each variable than regression analysis for the data collected in only two time points, and therefore minimizes the problem of measurement errors. This method has been adopted in other longitudinal studies (e.g., Nolen-Hoeksema et al. 2007) and experience-sampling studies (e.g., Moberly and Watkins 2008).

We repeated the analyses using as fixed factors the durations between each testing occasion and each time point at which the dependent variables were assessed (Times 2 and 3). Because the results were quite similar, we did not treat these variables as fixed factors, in order to reduce the number of predictor variables.

We repeated the same analyses with the RRS total scale placed by the brooding and reflection subscales. Finally, we analyzed linear mixed models in order to test whether depression, rumination, and each dimension of social problem solving predicted frequencies of interpersonal stressors experienced by participants during the next 4 weeks. In order to reduce the number of variables entered, we treated as fixed factors only initial interpersonal stressors and variables that were correlated with subsequent stressors.

## Results

Table 2 shows correlations between each measure at Time 1, and Table 3 shows correlations between each variable at Time 1 and Time 2, and those between Time 2 and Time 3. Table 4 shows the results of evaluating the linear mixed models that examined whether each variable predicted depression, rumination assessed with RRS total scale, and each dimension of social problem solving at the next time point. Rumination and impulsivity/carelessness style were predictive of more severe depression at subsequent time points. These findings suggested that if a person’s rumination and impulsivity/carelessness style scores were to increase by 1 *SD*, his or her depression scores would subsequently increase by 0.12 *SD* and 0.06 *SD*, respectively. Depression, positive problem orientation, and negative problem orientation predicted higher levels of subsequent rumination. That is, if a person’s depression, positive problem orientation, and negative problem orientation scores were to increase by 1 *SD*, his or her rumination scores would subsequently increase by 0.08 *SD*, 0.11 *SD*, and 0.14 *SD*, respectively. Rumination and avoidance style predicted higher levels of subsequent negative problem orientation, and positive problem orientation predicted lower levels of subsequent negative problem orientation. These results indicate that if a person’s rumination and avoidance style scores were to increase by 1 *SD*, his or her negative problem orientation scores would subsequently increase by 0.16 *SD* and 0.09 *SD* respectively, and if a person’s positive problem orientation scores were to increase by 1 *SD*, his or her negative problem orientation scores would subsequently decrease by 0.10 *SD*. Impulsivity/carelessness style predicted lower levels of rational problem solving assessed at subsequent time points. Impulsivity/carelessness style predicted higher levels of subsequent avoidance style, and positive problem orientation predicted lower levels of subsequent avoidance style, suggesting that if a person’s impulsivity/carelessness style and positive problem orientation scores were to increase by 1 *SD*, his or her avoidance style scores would subsequently increase by 0.06 *SD* and decrease by 0.10 *SD*, respectively. Positive problem orientation and impulsivity/carelessness style were predicted only by themselves at prior assessments.

**Table 2:** Correlations between variables at Time 1

	1	2	3	4	5	6	7	8	9
1. BDI-II	–								
2. Brooding	.48***	–							
3. Reflection	.24***	.44***	–						
4. RRS total	.54***	.87***	.70***	–					
5. Positive problem orientation	−.21***	−.08	.26***	.00	–				
6. Negative problem orientation	.48***	.58***	.14*	.53***	−.33***	–			
7. Rational problem solving	−.01	.28***	.40***	.34***	.45	.06	–		
8. Impulsivity/carelessness style	.13*	.03	.03	.07	.06	.19**	−.16**	–	
9. Avoidance style	.30***	.28***	.07	.31***	−.21***	.50***	.02	.35***	–
10. ISE	.40***	.35***	.23***	.38***	.04	.22***	.14*	.13*	.20**

*BDI*-*II* Beck Depression Inventory-Second Edition, *RRS* Ruminative Responses Scale, *ISE* Interpersonal Stress Event Scale

* *p* < .05; ** *p* < .01; *** *p* < .001

**Table 3:** Correlations between variables at Time 1 and Time 2, Time 2 and Time 3

	1	2	3	4	5	6	7	8	9	10
Correlations between each variable at Time 1 (left side) and Time 2 (upper side)										
1.BDI-II	.81***	.46***	.18*	.50***	−.28***	.40***	−.03	.12	.34***	.33***
2. Brooding	.44***	.68***	.29***	.63***	−.21**	.49***	.10	.04	.23**	.29***
3. Reflection	.28***	.28***	.66***	.51***	.11	.14	.27***	.02	.03	.22**
4. RRS total	.55***	.65***	.53***	.77***	−.14*	.48***	.21**	.06	.24**	.33***
5. Positive problem orientation	−.10	−.05	.23**	.03	.72***	−.36***	.39***	−.03	−.32***	.03
6. Negative problem orientation	.43***	.53***	.12	.47***	−.39***	.74***	−.04	.20**	.51***	.22**
7. Rational problem solving	.13	.17*	.32***	.30***	.38***	−.02	.71***	−.22**	−.11	.22**
8. Impulsivity/carelessness style	.19**	−.02	.01	.03	−.04	.13	−.25***	.67***	.29***	.08
9. Avoidance style	.29***	.29***	.05	.30***	−.30***	.51***	.01	.26***	.78***	.12
10. ISE	.44***	.39***	.26***	.40***	−.02	.24**	.20**	.22**	.25***	.61***
Correlations between each variable at Time 2 (left side) and Time 3 (upper side)										
1. BDI-II	.82***	.46***	.31***	.53***	−.09	.41***	.05	.10	.26**	.58***
2. Brooding	.46***	.73***	.34***	.69***	.03	.58***	.14	.06	.34***	.33***
3. Reflection	.27**	.39***	.80***	.60***	.13	.22**	.20*	.17*	.21**	.34***
4. RRS total	.56***	.70***	.56***	.78***	.06	.55***	.22**	.11	.37***	.44***
5. Positive problem orientation	−.18*	−.09	.13	−.01	.66***	−.33***	.21**	.15	−.24**	.06
6. Negative problem orientation	.38***	.58***	.25**	.55***	−.26**	.79***	.07	.14	.51***	.25**
7. Rational problem solving	.01	.18*	.30***	.28***	.36***	.09	.65***	−.12	.10	.33***
8. Impulsivity/carelessness style	.17*	.08	.15	.11	−.01	.17*	−.19*	.77***	.30***	.15
9. Avoidance style	.29***	.31***	.11	.32***	−.21**	.54***	−.00	.29***	.82***	.14
10. ISE	.35***	.29***	.30***	.35***	.15	.25**	.22**	.18*	.10	.74***

*BDI*-*II* Beck Depression Inventory-Second Edition, *RRS* Ruminative Responses Scale, *ISE* Interpersonal Stress Event Scale

* *p* < .05; ** *p* < .01; *** *p* < .001

**Table 4:** Linear mixed models with each variable assessed at the next time point as dependent variables

Independent variables	BDI-II			RRS total		
	*B*	*SE*	*t*	*B*	*SE*	*t*
Intercept	−3.90	1.23	−3.17**	4.22	1.94	2.18*
BDI-II	0.82	0.04	21.70***	0.13	0.06	2.19*
RRS total	0.08	0.03	2.76**	0.72	0.05	15.87***
Positive problem orientation	0.05	0.08	0.66	0.34	0.13	2.65**
Negative problem orientation	−0.02	0.08	−0.22	0.42	0.13	3.25**
Rational problem solving	0.03	0.08	0.41	0.03	0.13	0.21
Impulsivity/carelessness style	0.15	0.07	1.98*	−0.16	0.12	−1.36
Avoidance style	−0.04	0.08	−0.48	0.07	0.12	0.57

Independent variables	Positive problem orientation			Negative problem orientation		
	*B*	*SE*	*t*	*B*	*SE*	*t*
Intercept	2.84	0.68	4.16***	0.24	0.62	0.39
BDI-II	−0.02	0.02	−0.89	−0.02	0.02	−1.18
RRS total	0.01	0.02	0.38	0.06	0.01	4.01***
Positive problem orientation	0.74	0.05	16.41***	−0.11	0.04	−2.61**
Negative problem orientation	−0.06	0.05	−1.26	0.74	0.04	17.55***
Rational problem solving	0.05	0.05	1.02	0.02	0.04	0.57
Impulsivity/carelessness style	−0.01	0.04	−0.18	0.00	0.04	0.07
Avoidance style	−0.03	0.04	−0.72	0.10	0.04	2.51*

Independent variables	Rational problem solving			Impulsivity/carelessness style		
	*B*	*SE*	*t*	*B*	*SE*	*t*
Intercept	2.49	0.67	3.73***	0.95	0.66	1.45
BDI-II	−0.01	0.02	−0.54	−0.02	0.02	−0.74
RRS total	−0.01	0.02	−0.49	0.01	0.02	0.85
Positive problem orientation	0.02	0.04	0.52	0.06	0.04	1.40
Negative problem orientation	0.03	0.04	0.73	−0.00	0.04	−0.01
Rational problem solving	0.74	0.05	16.29***	−0.07	0.05	−1.60
Impulsivity/carelessness style	−0.09	0.04	−2.16*	0.75	0.04	18.64***
Avoidance style	0.05	0.04	1.21	0.05	0.04	1.32

Independent variables	Avoidance style		
	*B*	*SE*	*t*
Intercept	1.18	0.56	2.10*
BDI-II	−0.01	0.02	−0.77
RRS total	0.01	0.01	0.80
Positive problem orientation	−0.10	0.04	−2.57*
Negative problem orientation	0.01	0.04	0.34
Rational problem solving	0.04	0.04	1.02
Impulsivity/carelessness style	0.07	0.03	2.05*
Avoidance style	0.85	0.04	24.14***

*BDI*-*II* Beck Depression Inventory-Second Edition, *RRS* Ruminative Responses Scale

* *p* < .05; ** *p* < .01; *** *p* < .001

We repeated the linear mixed models using the brooding subscale, instead of the RRS total scale, as fixed factors and dependent variables. Although the results were quite similar to those for RRS total scale, a few differences emerged. First, brooding did not predict depression assessed at subsequent time points (*B* = 0.13, *SE* = .09, *t* = 1.44, *p* = .16). Second, with brooding as a dependent variable, depression, positive problem orientation, and negative problem orientation predicted higher levels of brooding 4 weeks later, but impulsivity/carelessness style predicted lower levels of future brooding (*B* = −0.08, *SE* = .04, *t* = −2.23, *p* = .03).

Next, we analyzed linear mixed models with the reflection subscale as fixed factors and dependent variable. The following differences from the analysis using the RRS total scale emerged. First, reflection did not predict depression assessed at subsequent time points (*B* = 0.12, *SE* = .09, *t* = 1.46, *p* = .15). Second, with reflection as a dependent variable, only negative problem orientation predicted higher levels of reflection 4 weeks later (*B* = 0.10, *SE* = .04, *t* = 2.77, *p* = .01). Third, refection did not predict negative problem orientation at subsequent time points (*B* = 0.08, *SE* = .04, *t* = 1.80, *p* = .08).

Finally, linear mixed models were analyzed to determine which variables predicted interpersonal stressors assessed at the next time point. Since initial depression, rumination assessed with the RRS total scale, negative problem orientation, rational problem solving, and interpersonal stressors assessed with the ISE were correlated with subsequent stressors (see Table 3), we used these variables as fixed factors. Table 5 displays the results. Depression and rational problem solving predicted increases in stressors 4 weeks later, indicating that if a person’s depression and rational problem solving scores were to increase by 1 *SD*, subsequently his or her interpersonal stressor scores would increase by 0.24 *SD* and 0.13 *SD*, respectively.

**Table 5:** Linear mixed models with Interpersonal Stress Event Scale assessed at the next time point as dependent variable

	*B*	*SE*	*t*
Intercept	3.76	0.89	4.23***
BDI-II	0.15	0.03	4.90***
RRS total	−0.01	0.02	−0.44
Negative problem orientation	−0.03	0.05	−0.60
Rational problem solving	0.17	0.06	3.13**
ISE	0.57	0.04	13.72***

*BDI*-*II* Beck Depression Inventory-Second Edition, *RRS* Ruminative Responses Scale, *ISE* Interpersonal Stress Event Scale

** *p* < .01; *** *p* < .001

## Discussion

The present study examined longitudinal associations among the various dimensions of rumination and social problem solving, and what dimensions of rumination and social problem solving intensified depression assessed 4 weeks later. The results partially supported our predictions that rumination, negative problem orientation, and avoidance style would be longitudinally associated with each other. Rumination assessed with the RRS total scale predicted higher levels of negative problem orientation. This effect is in line with experimental findings that induction of state rumination increases negative interpretations of problematic situations (Lyubomirsky and Nolen-Hoeksema 1995; Lyubomirsky et al. 1999). Importantly, the present study found that negative problem orientation increased rumination, but neither rumination nor negative problem orientation increased interpersonal stressors. Therefore, negative problem orientation may increase rumination without obvious environmental change, although it is not impossible that this problem orientation leads to stressors in domains other than the interpersonal. Although rumination has not been shown to lead to active problem solving (Lyubomirsky and Nolen-Hoeksema 1995; Lyubomirsky et al. 1999), it is plausible that individuals with increased negative problem orientation ruminate about the self and past problematic situations to obtain information related to current situations. Furthermore, rumination, but not negative problem orientation, predicted subsequent depression. During the vicious cycle of rumination and negative problem orientation, rumination worsens depression because of the negative valence of thoughts (Lyubomirsky et al. 1999).

The present results also indicated that the avoidance style increased negative problem orientation, again in the absence of environmental change. It is possible that a negligible attempt to solve a problem leads to negative interpretation of the situation, given that the individual maintains his/her attention on a problematic situation that does not change. However, neither rumination nor negative problem orientation predicted subsequent avoidance style, suggesting that rumination does not work to increase an avoidance-based problem solving style.

In line with our prediction, rumination did not predict subsequent impulsivity/carelessness style, and vice versa. These findings extend previous studies of concurrent associations between these variables (Hasegawa et al. 2015b, 2016). After controlling for the influence of initial depression, rumination and impulsivity/carelessness style were still predictive of more severe depression 4 weeks later. It is plausible that depressive rumination and an impulsive/careless problem solving style are independent processes that work to intensify depression. Impulsivity/carelessness style did not predict the frequency of subsequent encounters with interpersonal stressors, but because this problem solving style, unlike rumination, does not include negative thought, we think it is unlikely that it intensifies depression without any environmental change. It is possible that individuals with a higher level of impulsivity/carelessness style tend to make mistakes while solving problems in the academic or work domains.

Although the present study showed that rumination and impulsivity/carelessness style are independent processes, Valderrama et al. (2016) found positive associations between rumination and impulsivity. Of the four dimensions of the UPPS Impulsive Behavior scale (Whiteside and Lynam 2001), negative urgency, which represents the tendency to act in a rash manner when experiencing negative emotions, was positively associated with brooding (*r* = .29). On the other hand, performance on the stop signal task, a behavioral measure of the response disinhibition aspect of impulsivity, was not associated with rumination (Aker et al. 2014). Because impulsivity is assumed to consist of heterogeneous dimensions, including, but not limited to, an impulsivity/carelessness style, more studies are necessary to examine what dimensions of impulsivity are related to rumination.

While rumination predicted severity of subsequent depression, depression predicted higher levels of rumination at subsequent time points. Other longitudinal studies have similarly shown depressive symptoms to be significant positive predictors of subsequent rumination (Nolen-Hoeksema et al. 1999; Hasegawa et al. 2015a). A reciprocal relationship between rumination and depression was also suggested by an experience-sampling study that examined the association between state ruminative self-focus and negative affect (Moberly and Watkins 2008). It is plausible that “negative affect may result in a search for explanatory causes and trigger self-regulatory attempts to repair the negative mood and enact behavioral coping strategies” (Moberly and Watkins 2008, p. 321), while these processes are regarded as aspects of depressive rumination (Nolen-Hoeksema 1991).

Positive problem orientation predicted *higher* levels of subsequent rumination. This finding is surprising, because positive problem orientation is thought to enhance subsequent problem solving efforts (D’Zurilla et al. 2002). However, the positive association between positive problem orientation and future rumination is consistent with the suggestion of some researchers that problem solving intentions are the basis of prolonged rumination (Papageorgiou and Wells 2001a, b; Watkins and Baracaia 2001). It is possible that some individuals ruminate in an attempt to solve problems. However, because positive problem orientation was negatively related to concurrent depression (see Table 2) and predicted subsequent lower levels of negative problem orientation and avoidance style, it does not seem plausible that promoting positive problem orientation can increase rumination.

Because impulsivity/carelessness style reflects impulsive responses and rational problem solving reflects more thoughtful responses, a negative longitudinal association of impulsivity/carelessness style with rational problem solving is understandable. But although rational problem solving is thought to represent an effective approach to problem solving (D’Zurilla et al. 2002), the observed *positive* effect of rational problem solving on increased interpersonal stressors stands in contrast to social problem solving theory. The notion that rational problem solving assesses a dimension of effective problem solving is doubtful, because many studies have shown non-significant correlations between rational problem solving and depression measures (e.g., D’Zurilla et al. 1998; Anderson et al. 2011; Hasegawa et al. 2015b, 2016; see also D’Zurilla et al. 2002). It is possible that the rational problem solving subscale of the SPSI-R or SPSI-R:S reflects, at least in part, the use of maladaptive strategies that work to deteriorate interpersonal relationships. The rational problem solving subscale assesses not quality of problem solving but rather frequency of use of problem solving strategies. Some participants with higher scores on rational problem solving may engage in excessive reassurance seeking or information gathering from others, thereby irritating or alienating these social contacts.

Finally, we discuss findings from analyses that replaced RRS total scale with brooding and reflection subscales. Brooding did not predict depression assessed at subsequent time points. Hasegawa et al. (2013) previously showed that the RRS total scale had greater predictive power than did the brooding subscale for depression assessed 8 weeks later. The weak predictive power of the brooding subscale may be due to relatively weak internal consistency (*αs* ranged from .78 to .82) and a narrower range of subscale scores (*SDs* ranged from 3.92 to 3.96) than the RRS total scale (*αs* ranged from .91 to .93, and *SDs* ranged from 13.04 to 13.84; see Table 1). Next, impulsivity/carelessness style predicted future brooding. But since the predictive value of impulsivity/carelessness style was negative, this result does not contradict our proposal that rumination and impulsive/careless problem solving style exacerbate depression via relatively independent processes, or at least do not mutually enhance one another.

The analyses using the reflection subscale indicated that reflection represents a less maladaptive aspect of rumination. The reflection subscale predicted neither subsequent depression nor negative problem orientation and was itself not predicted by depression at previous time points. These results suggest that associations between reflection on the one hand and depression and negative problem orientation on the other are indeed weak. Reflection represents thought processes whose valence is less negative (see the definition and item content of the reflection subscale as described above). The less negative valence of thoughts may prevent a reciprocal cycle of reflection and depression and may not increase negative interpretations about problematic situations (i.e., negative problem orientation).

The present study is the first to show longitudinal associations among rumination, negative problem orientation, and avoidance style. In addition, depressive rumination and impulsivity/carelessness style appear to be independent processes that work to intensify depression. The present study was a three-wave longitudinal design that examined the predictive powers of each variable using linear mixed models. This analytic method produces relatively stable longitudinal associations between each variable, which minimizes the problem of measurement errors.

This study has two clinical implications. First, rumination is related longitudinally to negative problem orientation, and vice versa. Rumination and negative problem orientation are two major factors that intensify depression (Nolen-Hoeksema and Morrow 1991; Nolen-Hoeksema 2000; Spasojević and Alloy 2001; Anderson et al. 2011), and several cognitive behavioral approaches are available for intervention of both components. There are several empirically supported therapies that target maladaptive components of rumination. For example, rumination-focused cognitive behaviour therapy, developed by Watkins and his colleagues (Watkins et al. 2007) is an effective psychotherapy for alleviating rumination and depression in patients with medication-refractory residual depression (Watkins et al. 2011). Rumination is conceptualized as consisting of abstract, evaluative cognitive processing and avoidance behaviors. Rumination-focused cognitive behaviour therapy aims at replacing rumination with concrete, process-focused, and specific cognitive processing, as well as with more helpful approach behaviors.

On the other hand, problem-solving therapy (Nezu et al. 1989) is a well-established treatment intended to improve dimensions of social problem solving (Nezu et al. 2008, for review). By means of discussion, reverse role playing, and making lists of problems, therapists stimulate clients to use negative emotion as a means of identifying problems and to perceive their problems as controllable and a normal experience (Nezu et al. 1989). These procedures are focused on decreasing negative problem orientation and increasing positive problem orientation. Integration of these therapies to intervene in the vicious cycle of rumination and negative problem orientation may improve the effectiveness of depression treatments.

The present results also indicated that rumination and impulsivity/carelessness style are independent processes that serve to intensify depression. If this notion is true, the addition of techniques to target an impulsive/careless problem solving style may increase the effects of treatment for depression. In problem-solving therapy, therapists try to discourage clients from responding impulsively in problem solving situations and instead encourage them to engage rationally in the problem solving process (Nezu et al. 1989). However, there is no established therapy that targets reduction of impulsivity/carelessness style or impulsivity in general. Because depressive symptoms may be alleviated by decreasing the tendency for impulsivity/carelessness style (and impulsivity), it is important to develop such a treatment.

Several limitations of this study should be noted. The sample was composed of nonclinical university students. The assessments utilized were all self-report measures, and because the participants completed the questionnaires at their homes, it is unclear whether the participants accurately reported the day on which the questionnaires were completed (we could not conduct an online survey because Nihon Bunka Kagakusha Co., Ltd., prohibits online use of the Japanese version of BDI-II). Moreover, the intervals between the times at which participants completed the questionnaires varied somewhat, although the results did not change after controlling for this variable. Future studies should address these limitations to more clearly demonstrate longitudinal associations among depression, rumination, and social problem solving.
